# When the fever will not stop, stop the pills! A case report

**DOI:** 10.1590/1516-3180.2022.0401.R1.13032023

**Published:** 2023-12-04

**Authors:** César Ricardo Coimbra de Matos, Eduarda Maria da Conceição Sério Pereira Beirão, Rafael Simões Neves, António José Assunção, Rui Moreira Marques

**Affiliations:** I MD. Physician, General and Family Medicine, Personalized Care Unit, Tábua, Portugal.; II MD. Physician, General and Family Medicine, Personalized Care Unit, Tábua, Portugal.; III MD. Physician, General and Family Medicine, Personalized Care Unit, Tábua, Portugal.; IV MD. Physician, General and Family Medicine, Personalized Care Unit, Gouveia, Portugal.; V MD. Physician, Internal Medicine, Tondela-Viseu Hospital Center, Viseu, Portugal.

**Keywords:** Neuroleptic malignant syndrome, Antipsychotic agents, Fever, Drug-related side effects and adverse reactions, Risk factors, Neuroleptics, Adverse drug reactions, Autonomic dysfunction, Factor, risk

## Abstract

Neuroleptic malignant syndrome (NMS) is a neurologic emergency potentially fatal. This rare side effect is most commonly associated with first-generation antipsychotics and less frequently with atypical or second-generation antipsychotics. The diagnosis relies on both clinical and laboratory criteria, with other organic and psychiatric conditions being ruled out.

**CASE REPORT:**

A 39-year-old female patient, who is institutionalized and completely dependent, has a medical history of recurrent urinary infections and colonization by carbapenem-resistant *Klebsiella pneumoniae.* Her regular medication regimen included sertraline, valproic acid, quetiapine, risperidone, lorazepam, diazepam, haloperidol, baclofen, and fentanyl. The patient began experiencing dyspnea. Upon physical examination, she exhibited hypotension and a diminished vesicular murmur at the right base during pulmonary auscultation. Initially, after hospitalization, she developed high febrile peaks associated with hemodynamic instability, prompting the initiation of antibiotic treatment. Despite this, her fever persisted without an increase in blood inflammatory parameters, and she developed purulent sputum, necessitating antibiotherapy escalation. The seventh day of hospitalization showed no improvement in symptoms, suggesting NNMS as a differential diagnosis. All antipsychotic and sedative drugs, as well as antibiotherapy, were discontinued, after which the patient showed significant clinical improvement.

**CONCLUSION:**

Antipsychotic agents are commonly employed to manage behavioral changes linked to various disorders. However, their severe side effects necessitate a high degree of vigilance, the cessation of all medications, and the implementation of supportive care measures. A prompt and accurate diagnosis of NMS is crucial to alleviating the severe, prolonged morbidity and potential mortality associated with this syndrome.

## INTRODUCTION

Neuroleptic malignant syndrome (NMS) is an uncommon (incidence of 0.01% to 3.23%) but potentially lethal idiosyncratic reaction (**Table 1**) that may emerge in the aftermath of treatments with neuroleptics, demonstrating itself with symptoms ranging from altered consciousness to death.^1,2^

**Table 1.** Bibliography

Reference	Database	Search strategy	Data	Filter	Nº Results	Results
1	MEDLINE/PubMed	“neuroleptic malignant syndrome” [MeSH] AND “antipsychotic agents” [MeSH] AND “fever” [MeSH]	28-08-2022	2017–2022	26	1. Case Report: 13 2. Original article: 7 3. Narrative Review: 3
2	MEDLINE/PubMed	“neuroleptic malignant syndrome” [MeSH] AND “antipsychotic agents” [MeSH] AND “neuroleptics” [MeSH]	28-08-2022	2017–2022	131	1. Meta-Analysis: 3 2. Original article: 12 3. Narrative Review: 23
3	MEDLINE/PubMed	“neuroleptic malignant syndrome” [MeSH] AND “antipsychotic agents” [MeSH] AND “risk factors” [MeSH]	28-08-2022	2017–2022	11	1. Case Report: 1 2. Original article: 1 3. Narrative Review: 9
4	MEDLINE/PubMed	“neuroleptic malignant syndrome” [MeSH] AND “adverse drug reactions” [MeSH] AND “risk factors” [MeSH]	28-08-2022	2017–2022	4	1. Original article: 1 2. Narrative Review: 3
5	MEDLINE/PubMed	“neuroleptic malignant syndrome” [MeSH] AND “antipsychotic agents” [MeSH] AND “adverse drug reactions” [MeSH]	28-08-2022	2017–2022	26	1. Meta-Analysis: 1 2. Case Report: 11 3. Original article: 7 4. Narrative Review: 7
6	MEDLINE/PubMed	“neuroleptic malignant syndrome” [MeSH] AND “autonomic dysfunction” [MeSH] AND “fever” [MeSH]	28-08-2022	2017–2022	3	1. Original article: 1 2. Narrative Review: 2
7	MEDLINE/PubMed	“neuroleptic malignant syndrome” [MeSH] AND “antipsychotic agents” [MeSH] AND “drug-related side effects” [MeSH]	28-08-2022	2017–2022	7	1. Case Report: 2 2. Original article: 2 3. Narrative Review: 3
8	MEDLINE/PubMed	“neuroleptic malignant syndrome” [MeSH] AND “autonomic dysfunction” [MeSH] AND “fever” [MeSH]	28-08-2022	2017–2022	4	1. Narrative Review: 4
9	MEDLINE/PubMed	“neuroleptic malignant syndrome” [MeSH] AND “fever” [MeSH] AND “risk factors” [MeSH]	28-08-2022	2017–2022	3	1. Case Report: 2 2. Narrative Review: 2
10	MEDLINE/PubMed	“neuroleptics” [MeSH] AND “antipsychotic agents” [MeSH] AND “risk factors” [MeSH]	28-08-2022	2017–2022	812	1. Meta-Analysis: 45 2. Randomized Controlled Trial: 28 3. Narrative Review: 55

High-potency first-generation antipsychotics (FGAs) are associated with the highest incidence of NMS, e.g., haloperidol. Though second-generation antipsychotics (SGAs) have a lower incidence, low-potency FGAs have not been imputed alone in any case of NMS.^3^

Additional risk factors identified include pre-existing organic pathologies of the central nervous system, lithium treatment, infections, and the cessation of medication with anticholinergic properties or alcohol. Given the rising use of SGAs, it is crucial to remain cognizant of the associated risk of NMS.^4^

The diagnostic criteria for NMS encompass altered mental status, heightened muscle stiffness, fever, autonomic dysfunction, and analytical alterations such as elevated creatine phosphokinase (CPK). However, the presentation of NMS can vary, with some patients developing the syndrome without rigidity.^5-7^ Consequently, there is no specific test available for NMS, and diagnosis relies heavily on clinical suspicion.^8^

NMS complications can lead to multiple organ system failure, aspiration pneumonia, pulmonary embolism, disseminated intravascular coagulation, and persistent cognitive sequelae. These long-term cognitive effects are often the result of hypoxia and prolonged hyperthermia.^6^

While it is not always possible to scientifically validate treatment recommendations, the significance of prompt supportive care is universally accepted. Additionally, discontinuing the use of causative antipsychotics is crucial to reducing mortality rates.^9,10^

Significantly reducing the morbidity and mortality of this perilous condition can be achieved through the minimization of risk factors, early identification, and swift management. A multidisciplinary approach could potentially be the key to a successful outcome.^6^

Further research is urgently required to scientifically substantiate the pathophysiology of NMS and formulate evidence-based treatment guidelines.^9^

## CASE REPORT

This case report was approved on July 29, 2022 (#05, Ethics Committee of Tondela-Viseu Hospital Center, Viseu). A female patient, 39 years old, was institutionalized in a Continuing Care Unit, totally dependent on her daily life activities, usually conscious, reactive to stimuli, non-collaborating, and with periods of psychomotor agitation. Medical history included percutaneous endoscopic gastrostomy (PEG) and bladder catheter, mental retardation since childhood, epilepsy, recurrent urinary infections, and colonization by carbapenem-resistant *Klebsiella pneumoniae* (KPC). Her regular medications were sertraline 100 mg id, valproic acid 500 mg 3 id, quetiapine 100 mg 2 id, quetiapine 50 mg id, risperidone 2 mg 3 id, lorazepam 5 mg id, lorazepam 2,5 mg 2 id, diazepam 5 mg id, haloperidol oral solution, ipratropium/salbutamol 0.5/0.25 mg id, budesonide 200 mcg 2 id, acetylcysteine 600 mg id, baclofen 10 mg 3 id, lactulose id, ferrous sulfate 329.7 mg id and transdermal fentanyl 12.5 mcg/h. There was no recent history of recent dose changes, evidence of overdose, or the introduction of a new medication. There were no known drug allergies.

The patient was sent to the emergency service for dyspnea and desaturation (82% ambient air) and had no other symptoms (such as cough, fever on admission, nasal obstruction/rhinorrhea), having performed sputum culture 20 days earlier with isolation of *Proteus mirabilis* meropenem-sensible. On physical examination, she was prostrated, non-collaborating, with mucocutaneous pallor, presenting a PEG and bladder catheter, and without pitting edema in her lower extremities. She had a blood pressure of 88/61 mmHg, a pulmonary auscultation with a decreased vesicular murmur at the right base, and a cardiac auscultation without alterations. Blood analysis, urinary screening, and arterial blood gas tests were executed with no analytical changes. Chest X-ray with slight bilateral hilar enhancement and blood cultures without bacterial growth. A head computed axial tomography scan without contrast was performed for “mild signs of ischemic leukoencephalopathy. Mild ventricular enlargement, reflecting diminished encephalic volume and subcortical atrophy, was more than expected for the patient’s age. Minor old lacunar strokes in the right striatocapsular region”.

The patient was admitted to the hospital for oxygen therapy, presumed to be suffering from a respiratory infection caused by *Proteus mirabilis*. Treatment with amoxicillin-clavulanic acid was initiated.

On D2, the patient began experiencing a fever peak (39–40 °C), accompanied by episodes of psychomotor agitation and hemodynamic instability. This necessitated the use of peripheral cooling and antipyretics to manage the fever. Analytical control revealed no significant deviations from the previous day’s results. However, due to the rapid deterioration associated with hemodynamic instability, a decision was made to alter the antibiotic therapy, initiating meropenem.

On D4, the fever peaks (4–4 hours) persisted despite the administration of antipyretics, and analytically, the inflammatory parameters were not elevated. Blood analysis showed leukocytes 7.10 × 10^9/L, segmented neutrophils 79.3%, hemoglobin 11.5 g/dL, sodium 136 mmol/L, potassium 4.2 mmol/L, chloride 101.8 mmol/L, urea 30 mg/dL, creatinine 0.2 mg/dL, reactive C protein 1.0 mg/dL and CK 2,500 U/L. Lumbar puncture was not possible due to the patient’s instability and bone deformities of the lumbar spine. At that moment, the patient presented expectoration with a purulent appearance, and a microbiological examination was performed. Vancomycin and fluconazole were started for greater microbiological coverage.

In D7, there was no improvement in fever nor changes in clinical and analytical parameters (showed leukocytes 7.9 × 10^9/L, segmented neutrophils 76.2%, hemoglobin 11.4 g/dL, sodium 138 mmol/L, potassium 4.1 mmol/L, chloride 104.1 mmol/L, creatinine 0.4 mg/dL, reactive C protein 1.2 mg/dL, and CK 2,840 U/L).

NMS was suspected as a differential diagnosis, and all antipsychotics, sedative drugs, and antibiotherapy were suspended. After 24 hours, the patient presented a good clinical evolution with sustained apyrexia, which suggested this syndrome as the most likely diagnosis. Oxygen therapy was slowly withdrawn as the patient became eupneic without oxygen needs. The Psychiatric Team resumed and adjusted psychiatric medication 2 weeks after the event, maintaining only sertraline 100 mg id, valproic acid 500 mg 3 id and stopping all other psychiatric medications (quetiapine 100 mg 2 id, quetiapine 50 mg id, risperidone 2 mg 3 id, lorazepam 5 mg id, lorazepam 2.5 mg 2 id, and transdermal fentanyl 12.5 mcg/h), with favorable evolution and without new clinical worsening.

## DISCUSSION

When symptoms no longer present a logical explanation, it is imperative to pause and consider the patient at hand. In this woman’s case, overlooking the NMS hypothesis could have led to a fatal outcome, likely due to an iatrogenic cause. A high degree of clinical suspicion and meticulous anamnesis are essential to deducing a differential diagnosis, as demonstrated in this case.

The escalating prevalence of chronic diseases and polymedication underscores the growing need for drug deprescription. Equally crucial is the implementation of appropriate therapeutic management for each patient, a task in which family doctors play a pivotal role. In the differential diagnoses of any polymedicated patient, particularly those on a regimen of both traditional and atypical antipsychotics, the presence of NMS must be considered, as exemplified by our patient’s case.

The text also highlights the contemporary issues of quaternary prevention and deprescription, areas that frequently fall short in our clinical practice.

Our case diverges from typical NMS cases due to the absence of rigidity. The patient initially exhibited hypotension, psychomotor agitation, dyspnea, and an unexplained fever during hospitalization. These symptoms, while potentially indicative of other diagnoses, particularly infectious ones, were present in this case. The patient’s clinical improvement following the complete discontinuation of medication supported the provisional diagnosis of NMS.

## CONCLUSION

The concurrent utilization of multiple medications, known as “polymedication,” coupled with physiological alterations impacting pharmacokinetics and pharmacodynamics leads to an increased likelihood of adverse side effects.

The diagnosis of NMS is fundamentally clinical and necessitates a high degree of suspicion. The treatment is primarily supportive.

This case report underscores the significance of the prompt and precise diagnosis of NMS, which is crucial in reducing severe, prolonged morbidity and potential mortality.
